# Prediction of protein-protein interactions using point transformer and spherical Convex Hull graphs

**DOI:** 10.1016/j.csbj.2025.12.008

**Published:** 2025-12-16

**Authors:** David Arteaga, Maria Poptsova

**Affiliations:** International Laboratory of Bioinformatics, Institute of Artificial Intelligence and Digital Sciences, National Research University Higher School of Economics, Moscow, Russia

**Keywords:** Protein-protein prediction, Point transformer, Graph neural networks, Large language models, Geometric deep learning, Spherical convex hull methods, PINDER

## Abstract

Accurate predictions and large-scale identification of protein-protein interactions (PPIs) are crucial for understanding their inherent biological mechanisms and protein functions in virtually all biological processes. Nowadays, graph-based deep learning models have made significant contributions in modeling proteins with physicochemical and geometric features. However, most of these models rely on conventional graph construction methods, such as radial cutoff or k-nearest neighbor (k-NN), which often produce sparse and weakly connected graphs, limiting the ability of neural networks to exploit the spatial relationships between nodes. To address this, we introduce PT-PPI, a geometric deep learning framework that combines protein surface point clouds with geometric graphs. Protein surfaces are encoded as oriented point clouds enriched with geometric features, then transformed into sparse, well-connected graphs using the hyperparameter-free Spherical Convex Hull (SCHull) method. These graphs are processed by a Point Transformer network, with representations coupled to ProstT5 sequence embeddings. Evaluations on the PINDER dataset show that PT-PPI surpasses LLM-based (D-SCRIPT), graph-based (GCN, GAT, Struct2Graph), and hybrid sequence-structural-based models (SpatialPPIv2). Ablation studies confirm the complementary value of surface geometry and sequence information, demonstrating that geometric deep learning on protein surfaces and point cloud representations offers a promising approach that opens the doors for further research on large-scale interactome mapping and the understanding of protein function.

## Highlights

• PT-PPI is a novel geometric deep learning framework for protein-protein interaction (PPI) prediction.
• Implements the Spherical Convex Hull (SCHull), a hyperparameter-free method for constructing sparse and connected geometric graphs.
• Represents protein surfaces as oriented point clouds enriched with geometric and chemical features.
• Leverages a Point Transformer network to capture complex spatial relationships within protein structures.
• Achieves state-of-the-art performance, outperforming existing sequence-based, graph-based, and hybrid models on the PINDER benchmark.
• Ablation studies confirm the complementary value of surface geometry and sequence embeddings for robust prediction.

## Graphical Abstract

**Figure:**
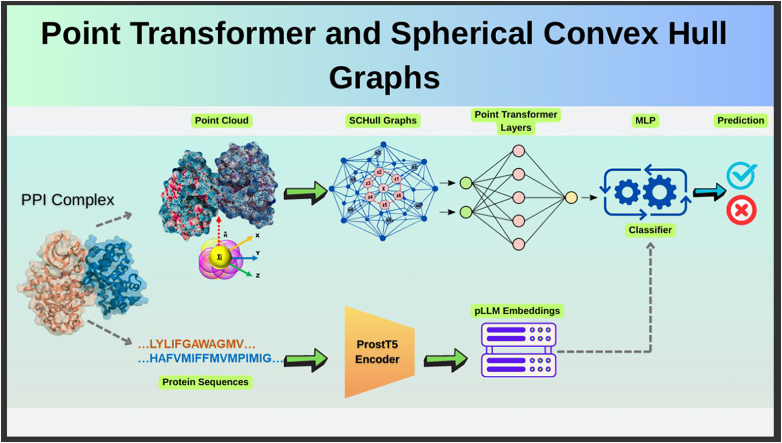

## 1 Introduction

Protein-protein interactions (PPIs) are central to biological processes, disease mechanisms, and therapeutic discovery [1], [2], [3], [4], [5]. Deep learning (DL) methods have demonstrated high efficiency by reducing experimental costs and time while enabling the analysis of extensive datasets generated through high-throughput procedures [6], [7], [8], [9]. While deep learning models using 3D structural data have advanced PPI prediction, the representation of protein structures remains a key challenge.

Most current methods represent proteins as graphs, where nodes correspond to atoms or residues and edges are defined by spatial proximity using fixed distance cutoffs. This approach requires a trade-off between connectivity and sparsity: overly dense graphs become computationally expensive, while overly sparse graphs risk losing critical structural information. In contrast, point clouds—unstructured sets of points in 3D space—provide an alternative representation that balances geometric fidelity with computational efficiency.

Originally developed for 3D computer vision tasks such as autonomous driving [10], [11], [12], 3D reconstruction [13], [14], [15], [16], [17], augmented reality [18], [19], and geospatial surveying [20], [21], point cloud methods have recently been adapted to protein structure analysis. In computational biology, point clouds enable faster processing and greater memory efficiency by directly representing molecular surfaces. Pioneering models include dMaSIF [22], which processes molecular surfaces as point clouds, and SurfaceID [23], a PointNet++-based model that predicts binding affinity from protein surface point clouds [24]. These have been followed by specialized methods for tasks such as protein docking evaluation (PointD [25]), PPI prediction (SurfPro-NN [26], PCLT-PPI [27]), ligand-binding site prediction [28], and atom-level interaction identification [29].

Recent advances in PPI prediction have increasingly promoted multimodal approaches that integrate graph neural networks, transformers, and protein language models. Methods such as SpatialPPIv2 [30], GAT/GCN [31], Struct2Graph [32], and GSMFormer-PPI [33] represent proteins as graphs with edges defined by distance thresholds. While effective, these approaches remain limited by their reliance on arbitrary connectivity cutoffs, which can omit geometrically meaningful long-range interactions or introduce unnecessary computational overhead.

To address these limitations, we introduce PT-PPI (Point Transformer for Protein-Protein Interactions), a framework that bridges the efficiency of point clouds with the relational learning of graph-based models. Unlike conventional GNNs, PT-PPI employs the parameter-free Spherical Convex Hull (SCHull) algorithm [34] to construct sparse yet fully connected geometric graphs directly from protein surface point clouds, avoiding predefined distance thresholds. These graphs, enriched with surface normals and geometric features, are processed by a Point Transformer network to capture complex spatial relationships, and the resulting features are combined with protein sequence embeddings for final interaction prediction.

The main contributions of this research are as follows.

1. A demonstration of the critical role of protein surface representation methods for PPI prediction, showing that the choice of structural representation significantly impacts model performance.
2. A novel representation of protein surfaces as oriented point clouds incorporating both unit normal vectors and key geometric features.
3. A method for constructing sparse, connected geometric graphs from these point clouds using the SCHull method, where nodes represent points with their associated geometric features.
4. The PT-PPI framework, which leverages a Point Transformer network to capture complex spatial relationships within the geometric graphs, derived from the oriented point cloud.

## 2 Methods

### 2.1 Dataset

In this research, we used the high-quality selection of dimer protein structures present in the Protein Interaction Dataset and Evaluation Resource (PINDER)[35]. It includes both experimentally resolved *holo* and *apo* structures, as well as computationally predicted complexes. A total of 56,177 protein complexes were used for training, validating, and testing our model. To ensure fairness, the same splits were used for training and evaluating all models employed for comparative analysis of our model’s performance against other state-of-the-art models, to evaluate the performance of the baselines, and in the ablation studies.

***Generation of Splits in PINDER.*** One of the main characteristics of PINDER is the high-quality organization of the dimeric systems. PINDER contains well-selected and filtered partitions for training, validating, and testing ML models. The potential leakage between training/validation and test sets is reduced by the implementation of a splitting algorithm focused on calculating the interface and sequence similarity of the protein structures. For a protein structure to be clustered into a split, the algorithm inspects several structural and sequential scores, such as the local Distance Difference Test score (lDDT), considering a threshold of 0.55 on the alignment graph, and the MMseqs2 [36] sequence identity score, considering a threshold of 30 %. These scores ensure that the test and validation splits contain protein structures that were completely excluded from the training set. Additionally, the benchmark studies of the PINDER dataset on the state-of-the-art docking model DiffDock-PPI [37] demonstrated that the clustering of proteins by interface similarity allows maximizing the diversity of the test set while minimizing the redundancy of proteins. For a detailed description of the algorithm, we recommend checking the original work of Kovtun and co-authors [35].

***Generation of Samples from PINDER Splits.*** For sampling the protein structures that were used in this study, we implemented the methodology developed by Hu and Ohue [30]. First, we downloaded all the PINDER complexes from the official repository. Using the scripts provided by Hu and Ohue [30], we extracted the sequences and coordinates of all the chains. To standardise the selection of complexes, we have chosen dimers where the Euclidean distance among residues is less than 8Å, and the length of their sequences varies between 35 and 300 amino acids. This filter allows selecting protein structures that offer meaningful information, such as sequential and structural context.

The negative samples were obtained through a random selection of potentially non-interacting proteins from the overall protein samples, followed by a cross-validation of those selected pairs using the BioGRID [38] database (v. 4.4.243, March 2025), to ensure that all the possible known interacting protein pairs were excluded. This is a technique frequently used by other authors due to the lack of experimentally curated negative samples with available and usable 3D structures. It aims to guarantee the scalability of protein pair selection through large data volumes [30]. Table 1 summarizes the datasets used in this study and their corresponding sizes.

**Table 1:** Datasets used in this research.

Split	Class	Source	Size
Train set	Positive	Pinder Train	50,060
	Negative	Random sampling	50,060
Validation set	Positive	Pinder Validation	1252
	Negative	Random sampling	1252
Test set	Positive	Pinder Test	1180
	Negative	Random sampling	1180

### 2.2 Protein surface feature extraction

We have implemented the fast-sampling algorithm developed by Sverrisson and co-authors [22] to calculate and generate protein surfaces on-the-fly from the atomic point cloud without relying on precomputed mesh structures. For computational efficiency, we used the KeOps [39] library within PyTorch to rapidly calculate the chemical and geometric features for each point.

***Chemical Features***. The protein surface was modelled by a cloud of atoms defined as a set $\{a_{1},\ldots ,a_{A}\}\in R^{3}$. This cloud is characterised by the presence of 22 atom types that were encoded as one-hot vectors $\{t_{1},\ldots ,t_{A}\}\in R^{22}$. The surface of the protein is then represented as an oriented point cloud $\{x_{1},\ldots ,x_{N}\}\in R^{3}$ that also contains unit normals ${\hat{n}}_{1},\ldots ,{\hat{n}}_{N}\in R^{3}$. Each point in the oriented cloud is associated with a feature one-hot vector. These vectors are then linearly mapped through an MLP layer that outputs a chemical feature vector $C_{i}\in R^{22}$. The atom types considered for the chemical features are provided in Table 2.

**Table 2:** Chemical and geometric features of the point cloud representation.

Feature type	Size	Description
**Chemical features**		
Atom types	22	H, Li, C, N, O, Na, Mg, P, S, K, Ca, Mn,
		Fe, Co, Ni, Cu, Zn, Se, Sr, Cd, Cs, Hg
**Geometric features**		
Mean	5	Scales from 1 Å to 10 Å
Gaussian	5	Scales from 1 Å to 10 Å

***Geometric Features***. For each point in the oriented point cloud $\{x_{1},\ldots ,x_{N}\}\in R^{3}$, we computed the Mean ($K_{\sigma ,i}$) and Gaussian ($H_{\sigma ,i}$) curvature as follows: $K_{\sigma ,i}=det(S_{\sigma ,i})$ and $H_{\sigma ,i}=trace(S_{\sigma ,i})$, where $S_{\sigma ,i}$ represents a 2 × 2 shape operator at point $x_{i}$, and σ∈[1,2,3,5,10] corresponds to the variation in the radius of the Gaussian windows used to compute the local curvature of each point by the implementation of quasi-geodesic convolutions and quadratic functions [40]. The variation of the Gaussian radii allows the extraction of geometric features at different scales, which can result in significantly informative features that characterise protein interactions at different levels of structural detail. Mean and Gaussian curvatures were computed at five different scales, resulting in feature vectors of dimensions $K_{i}\in R^{5}$ and $H_{i}\in R^{5}$, respectively. Once the chemical and geometric feature vectors were calculated, we concatenated them to form a unified representation within a shared feature space of dimension $\in R^{32}$. The complete set of initial feature dimensions for each point in the oriented point cloud is summarised in Table 2.

### 2.3 Graph construction

***Protein Graph Construction with Radial Cutoff***. The 3D protein structures, defined by the sequence of amino acids, are indispensable input data for training DL models, as they dictate the conditions that set the interactions with other molecules and ensure their functional roles [41]. These structures can be represented by a graph G=(V,E,X,E), where V and E denote the vertices and edges with their respective node (X) and edge (E) feature matrices. Naturally, there are multiple methodologies for creating protein graphs, with the most common being the radial cutoff and k-NN-based methods [41].

**Fig. 1:**
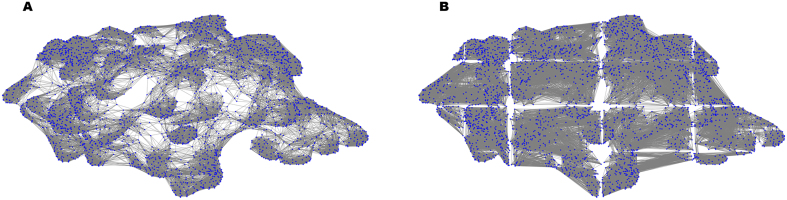
Radial cutoff graphs, r=3 and r=10. Protein 7v31, chain RA1, obtained using pyplot and network libraries, node size parameter = 10. A. Graph constructed with a radial cutoff of 3 Å. B. Graph constructed with a radial cutoff of 10 Å.

Several studies [30], [31], [42] have implemented methods for constructing graphs that represent the atomic structure of the protein [43]. However, determining the number of connections per atom is not a trivial task, and techniques like pruning, or removing edges of distant atoms, are often applied to regularize the complexity of the graphs. For example, setting a large cutoff threshold can ensure that graph nodes are well-connected; however, for large proteins, this may generate excessively dense structures that require high computational resources. In contrast, restricting the number of connections per node reduces graph density but risks creating disconnected components, thereby diminishing the amount of structural information available [44]. Graph connectivity and sparsity are thus critical factors that strongly influence the performance of graph neural networks (GNNs) in tasks such as PPI prediction. Prior studies [45] have demonstrated that insufficiently connected and sparse input graphs can negatively impact the computational efficiency and information flow throughout the network. Examples of protein graphs constructed with different radial cutoffs are presented in Fig. 1.

**Fig. 2:**
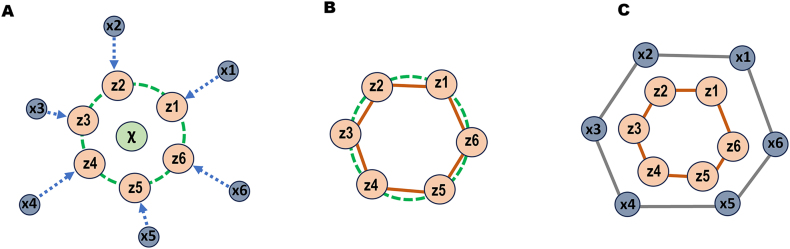
Graph construction using the SCHull method. A. For a given point cloud, $X_{i}$, we compute a unit sphere (green dashed line) centred at X. The projection of each $X_{i}$ onto this sphere results in the projected points $Z_{i}$. B. A convex hull and its corresponding edges (orange lines) are constructed from all $Z_{i}$ points on the sphere. C. The edges of the convex hull define the graph structure for the original point cloud. (For interpretation of the references to colour in this figure legend, the reader is referred to the web version of this article.)

***Spherical Convex Hull (SCHull) Method***. In this study, we employed the hyperparameter-free SCHull method [34] to construct molecular graphs (Fig. 2). SCHull works on spatially oriented points by first projecting them onto a unit sphere centred at the centroid of the point cloud. Then, it constructs a convex hull for the set of projected points. This convex hull, derived from non-coplanar points, is a polyhedral graph on the unit sphere whose vertices are a subset of the projected points, and whose edges define the hull. Once the convex hull is computed, the SCHull algorithm defines an edge between two original points only if the convex hull contains an edge connecting their corresponding projections. These relationships between points are stored as an adjacency matrix (*edge index*). This approach offers two benefits: it generates connected and sparse graphs and preserves the 3D arrangement of point clouds. Additionally, by edge distances and dihedral angles, SCHull provides sufficient geometric information for graph neural networks (GNNs) to infer node configurations while minimizing information loss.

The geometric graphs are constructed using the oriented point representation $\{x_{1},\ldots ,x_{N}\}\in R^{3}$ derived from the dMaSIF [22] framework. This point cloud can alternatively be represented as a set ${\left\{x_{i}\right\}}_{i=1}^{m}$ or as a spatial arrangement of nodes $V,X=[x_{1},x_{2},\ldots ,x_{m}]$. Each node contains an associated feature vector $f_{i}\in R^{n_{f}}$. Thus, the final graph will be defined as follows: G=(V,E,X), where V,E,X,F introduce a graph structure by connecting nodes using edges in the set E to include relationships between points in (V,X). $F=[f1,\ldots ,f_{m}]$ represents node features. Theoretical fundamentals of this approach are detailed in the original work of Wang and co-authors [34].

### 2.4 Point transformer

**Fig. 3:**
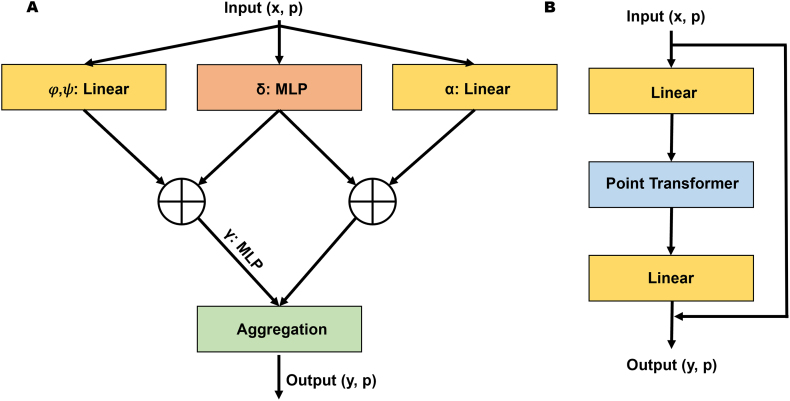
Point transformer architecture. A. Point transformer layer. B. Point transformer block.

The Point Transformer (PT) architecture consists of self-attention layers designed to process point cloud features and construct self-attention networks aimed at solving complex tasks such as object part or scene segmentation and classification [44], [46], [47], [48], [49]. PT extracts local and global features by implementing the well-known attention mechanism [50], which can capture spatial point relations and shape information. The self-attention mechanism is a set operator that is invariant to the permutation and cardinality of the input data, making it suitable for processing point cloud data [44]. The architecture of one PT layer and PT block is presented in Fig. 3.

**Fig. 4:**
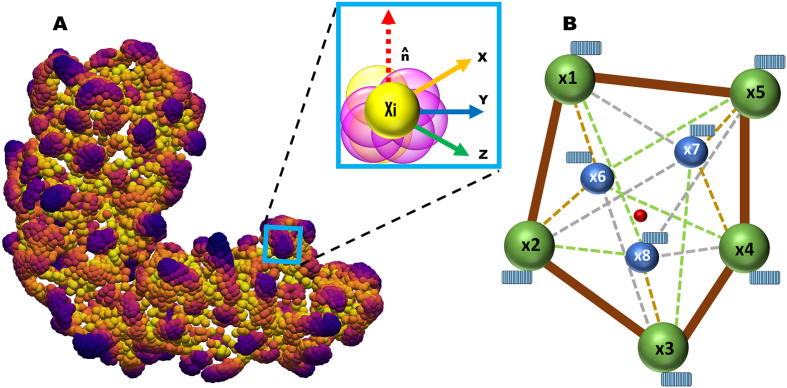
Point cloud representation and geometric graphs. A. Point cloud representation: X, Y, Z are the coordinates of each point $X_{i}$. B. Geometric graphs with $X_{1}\ldots \;X_{5}$ as nodes. Blue rectangles are 32D feature vectors associated with each node. (For interpretation of the references to colour in this figure legend, the reader is referred to the web version of this article.)

We computed geometric graphs for each pair of proteins involved in the complex, commonly called ligand (L) and receptor (R). These paired graphs and node features are the input of the point transformer layers. Fig. 4 shows a representation of the point cloud data, its features, and the geometric graph.

***Input Feature Encoding***. This module was designed to handle the geometric graphs generated by the implementation of the SCHull algorithm and the further incorporation of their node and edge features into the PT layers. SCHull outputs geometric graphs with three features: *x* (node features), *pos* (3D coordinates), and the *edge index* (adjacency matrix that defines the edges and the neighbouring structure). These features define the final graph G=(V,E,X), as previously mentioned.

The incorporation of the graph features into the PT-PPI architecture consists of the following steps:

Firstly, each node feature $x_{i}$ is projected into a higher-dimensional space H via:

(1) $$h_{i}^{\left(0\right)}=dropout(\sigma (LayerNorm(W_{in}xi))),$$

where $W_{in}\in R^{32xH}$ is a learnable projection matrix, σ denotes the ReLU activation function, and $x_{i}\in R^{Nx32}$ are the input node features, N being the number of nodes. Then, the positional encoding δ incorporates the global spatial information (*pos*) between neighbouring points $p_{i}$ and $p_{j}$ using the 3D coordinates as follows:

(2) $$\delta_{i,j}=h_{\Theta }(p_{i}-p_{j})$$

Here, $h_{\Theta }$ denotes an MLP with two linear layers that project the coordinates to the hidden dimensions. Hence, the final positional and node embeddings will be defined by $h_{i}^{\left(0\right)}\leftarrow h_{i}^{\left(0\right)}+\delta_{\left(i,j\right)}$.

***Point Transformer Layers***. The core of our model consists of two PT layers to capture local geometric context and relational dependencies among the nodes and their associated features. From the node feature encoding module, the PT layers receive $h_{i}^{\left(0\right)}$, the 3D coordinates (*pos*), and the *edge index*. Using these graph features, the PT layers compute pair-wise attention between neighbouring nodes.

We implemented the *PointTransformerConv* layer available in PyTorch Geometric [51]. For a node i, a PT layer outputs:

(3) $$x_{i}^{{\;}^{\prime }}=\sum_{j\in N(i)}\alpha_{i,j}(W_{3}x_{j}+\delta_{ij})$$

Here, the attention coefficient is computed as:

$\alpha_{i,j}=softmax(\gamma_{\Theta }(W_{1}x_{i}-W_{2}x_{j}+\delta_{ij}))$, where $\gamma_{\Theta }$ denotes an MLP.

$W_{1},W_{2},W_{3}$ are learnable projection matrices. After each transformer layer, it outputs:

$h_{i}^{\left(l+1\right)}\leftarrow dropout(\sigma (h_{i}^{\left(l+1\right)}))$.

Inside every PT layer, for each edge (i,j) defined by the *edge index* from SCHull, it computes a relative positional vector $\Delta_{ij}=p_{i}-p_{j}$ similarly to how the positional encoder δ was computed in the Input Feature Encoding module. Then each layer computes attention weights $\alpha_{i,j}$ by combining the node features and the positional encodings $\Delta_{ij}$. These calculations ensure that each point attends only to its neighbours defined by the *edge index* from SCHull. Consequently, the update is done by aggregation of neighbouring messages weighted by $\alpha_{i,j}$. In this way, the edges defined by the *edge index* regulate message passing, attention computation, and neighbourhood structure throughout the PT layers.

Finally, we applied mean pooling, after the last transformer layer, to aggregate the node embeddings into a fixed-size representation for each protein structure:

$g_{\text{mean}}=\frac{1}{\left|N_{c}\right|}\sum_{i\in N_{c}}h_{i}^{\left(L\right)}$, where $N_{c}$ corresponds to the node embeddings of each protein chain implicated in the paired complex.

**Fig. 5:**
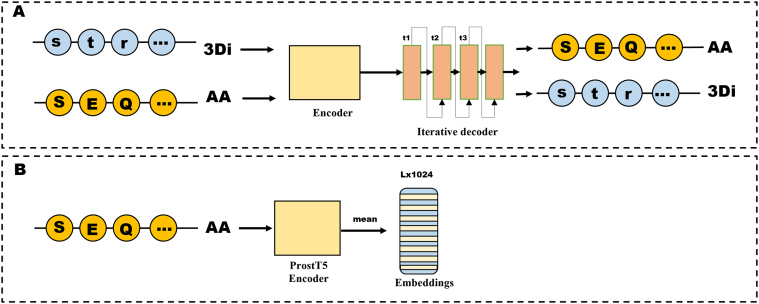
Generation of sequence embeddings using ProstT5. A. Illustration of the encoder-decoder architecture of ProstT5. B. Implementation of the ProstT5 encoder outputting a vector of dimension L×1024.

***Sequence Embeddings***. In addition to the protein surface features, we used the Protein Structure-Sequence T5 (ProstT5) [52] model to obtain the mean representation of the protein sequences. In essence, the ProstT5 is characterised by its ability to extract features from 3D structures and translate from sequences to structures and from structures to sequences. We used the model as an encoder implementing its half-precision (f16) mode, including batching. As input, it takes a sequence of amino acids of length L, and outputs its corresponding mean representation in the form of a vector of dimension (L, 1024). Each protein sequence embedding $s\in R^{\left(Lx1024\right)}$ is projected into the same hidden space as the geometric features after applying mean pooling. This is performed by a two-layer MLP. Fig. 5 shows the procedure applied by the model for obtaining the amino acid sequence representations.

### 2.5 PT-PPI model implementation

PT-PPI model was implemented using the open-source frameworks PyTorch and PyTorch Geometric [51]. The Point Transformer architecture followed the original implementation proposed by Zhao and co-authors [44], specifically, employing the PointTransformerConv layer from PyTorch Geometric. Hyperparameters, including the number of layers, were optimised empirically through controlled experiments using the same architecture and dataset. Benchmark models were obtained directly from their respective GitHub repositories, and their implementation adhered to the authors’ guidelines and specifications. Oriented point cloud representations were generated following the methodology of dMaSIF [22], complemented with additional preprocessing steps described by Hu and co-authors [40]. Geometric graphs were constructed using scripts provided by the SCHull authors [34]. Training was conducted for up to 80 epochs with early stopping (patience of 15 epochs) based on validation loss. To reduce overfitting, a dropout rate of 0.2 was applied after each layer. The optimisation was performed using the Adam optimiser with a learning rate of $1e^{-}4$, and a batch size of 32. This configuration was selected to balance training stability, computational efficiency, and generalization performance on our selected dataset.

## 3 Results and discussion

### 3.1 PT-PPI model: point transformer and spherical convex hull graphs

**Fig. 6:**
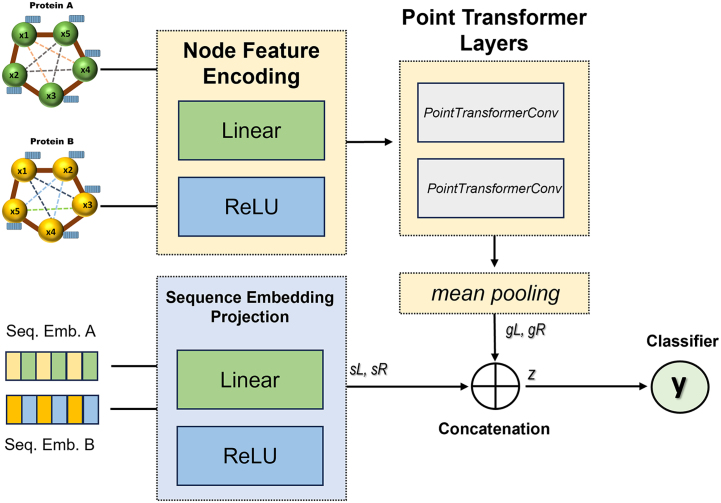
Architecture of PT-PPI model. The pooled and concatenated geometric features are represented by $g_{L},g_{R}$. The projection of the sequence embeddings is described by $s_{L},s_{R}$.

Fig. 6 illustrates the architecture of the PT-PPI model (see Methods for details). PT-PPI takes as input paired geometric graphs and their corresponding protein sequence embeddings. The nodes and their associated features are processed by the node feature encoding and point transformer modules, yielding a pooled vector that captures both local and global chemical as well as geometric features of the protein surface. Parallelly, ProstT5 sequence embeddings are processed by the sequence embedding projection module before being concatenated with the pooled geometric vectors. This architecture allows the model to learn complementary information from both geometric and sequential features before passing these vectors to the predictor.

### 3.2 Performance comparison with previous works

**Fig. 7:**
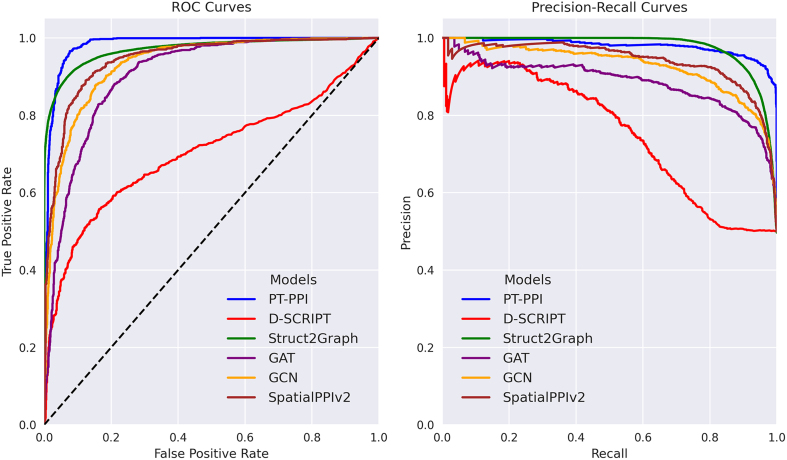
Performance evaluations of different models. A. Receiver operating characteristic (ROC) curves. B. Precision-recall (PR) curves.

The performance of the PT-PPI model was benchmarked against SOTA models from the field (Fig. 7). We divided these models into three groups. The first group includes models that use only the primary sequences of proteins as input for training and making predictions. Here, we included D-SCRIPT [53], a DL model that predicts PPIs using protein sequences to generate intermediate representations that capture the structural mechanism of interaction by using a pre-trained language model (Bepler & Berger’s model [54]). The prediction of interaction is based on the structural compatibility or contact map of the pair. This model consists of two main steps. During the first step, it generates the protein sequence representations for each protein separately, and in the second step, it estimates the likelihood of interactions based on these high-dimensional representations.

Our second group includes graph-based models that incorporate 3D structural information, residue proximity, and sequence embeddings for residue-level protein graph construction. We evaluated the GCN and GAT architectures proposed by Jha and co-authors [31]. These approaches use a graphical representation of the protein structure with residues as nodes. Each node was enriched with sequential embeddings obtained through the implementation of pre-trained language models, demonstrating that the sequence representations can be informative features that should be considered in DL models for predicting PPI. Additionally, we evaluated Struct2Graph [32], a DL model that creates a graph-based representation of the proteins using the 3D coordinates of atoms and applies multiple GCNs with weight sharing to generate relevant graph embeddings. Then, it extracts the relevant graph features of the query proteins by using a mutual attention network. These features are then concatenated and passed to a feed-forward neural network for inferring PPIs.

The third group includes the SpatialPPIv2 [30], a multimodal approach that uses protein-based language models and the PINDER dataset to obtain sequence embeddings and capture residue-level relationships via GAT networks and the attention mechanism. SpatialPPIv2 constructs graphs from adjacency matrices and amino acid sequence embeddings. These graphs are then updated by four GAT layers, and their output is pooled by chain to extract the overall graph features of the protein pairs. Finally, the prediction of interaction is performed through a fully connected layer.

All the models were trained, validated, and tested on the same dataset and splits as PT-PPI and compared using binary classification metrics such as accuracy, precision, recall, F1 score, ROC AUC, and PR AUC. All the parameters used for training and evaluating these models can be found in the Supplementary Materials.

Table 3 summarizes the results of a 3-fold cross-validation with random seeds. The primary purpose of the cross-validation is to measure the generalization capabilities of the model. In this case, the training set was randomly partitioned into three subsamples of equal size. Of these three subsamples, a single subsample is retained as the validation data for testing the models, and the remaining two subsamples are used as training data. We ran 3-folds for practical purposes and due to the size of the training set, which reaches around 100 k samples. Cross-validation with the D-SCRIPT [53] model was not possible because we used the pre-trained model that the authors provided on the official site.

**Table 3:** Averaged metrics and standard deviation across folds. Best results are in bold.

Model	Accuracy	Precision	Recall	F1 Score	AUROC	AUPRC
SpatialPPIv2 [30]	0.938 ± 0.0020	0.931 ± 0.0035	0.945 ± 0.0030	0.938 ± 0.0021	0.983 ± 0.0008	0.981 ± 0.0010
GAT [31]	0.928 ± 0.0025	0.918 ± 0.0040	0.934 ± 0.0038	0.926 ± 0.0023	0.979 ± 0.0010	0.976 ± 0.0012
GCN [31]	0.922 ± 0.0030	0.909 ± 0.0042	0.930 ± 0.0040	0.919 ± 0.0025	0.975 ± 0.0013	0.972 ± 0.0015
Struct2Graph [32]	0.915 ± 0.0020	0.924 ± 0.0120	0.903 ± 0.0120	0.913 ± 0.0010	0.970 ± 0.0000	0.969 ± 0.0010
PT-PPI	**0.966**± 0.0018	**0.945**± 0.0036	**0.988**± 0.0018	**0.966**± 0.0019	**0.994**± 0.0001	**0.994**± 0.0007

Table 4 presents performance metrics of our PT-PPI model and other models on the test set. It can be seen that PT-PPI outperformed all other models in all the evaluated metrics. These results indicate a highly reliable discrimination capability between interacting and non-interacting protein pairs.

**Table 4:** PPI models’ comparison on the test set. Best results are in bold.

Model	Accuracy	Precision	Recall	F1-score	AUROC	AUPRC
**Sequence-based**						
D-SCRIPT [53]	0.591	0.936	0.196	0.325	0.704	0.751
**Multimodal-based**						
SpatialPPIv2 [30]	0.868	0.884	0.847	0.865	0.935	0.936
**Graph-based**						
GAT [31]	0.835	0.807	0.881	0.842	0.904	0.885
GCN [31]	0.862	0.852	0.877	0.864	0.933	0.927
Struct2Graph [32]	0.914	0.924	0.902	0.913	0.970	0.969
**Our model**						
PT-PPI	**0.940**	**0.937**	**0.943**	**0.940**	**0.983**	**0.979**

In addition, in Table 5 we provide a comparison of the reported PT-PPI performance metrics with the reported metrics in the literature of our recently developed GDL-based model GSMFormer-PPI [33]. GSMFormer-PPI is a multimodal DL model that integrates surface, graph, and sequence modalities at the residue level through transformer encoders and linear projectors for predicting PPI with high accuracy. In this approach, the protein surfaces are described by geometric fingerprints delivered after pre-processing the structures with the MaSIF [55] framework. The graphs are formed by encoded nodes contextualized with sequence embeddings. These graphs are processed by GCN networks. The integration of surface and graph modalities was achieved through the implementation of projection layers and the self-attention mechanism of transformer encoders. Due to the high computational cost, it was challenging to evaluate GSMFormer-PPI on the same test as PT-PPI, which is why we present the results in a separate table. Although GSMFormer-PPI and PT-PPI differ in their architectural design, both models share an underlying aspect, the importance given to the 3D structure, the geometry, and physicochemical properties of the protein surfaces, demonstrating that this information should not be overlooked in future multi-modal architectures.

**Table 5:** Performance of the PT-PPI model vs GSMFormer-PPI.

Model	Accuracy	Precision	Recall	F1 Score	AUROC	AUPRC
GSMFormer-PPI [33]	0.963	0.957	0.974	0.965	0.989	0.991
PT-PPI	0.940	0.937	0.943	0.940	0.983	0.979

A comprehensive analysis of the results revealed that sequence-based methods, such as D-SCRIPT, exhibited the lowest performance among the evaluated models. Although it has a moderate ability to discriminate between classes and robustness in terms of class imbalance, D-SCRIPT fails when it comes to accurately predicting new protein pairs, where PT-PPI and other evaluated models demonstrate a substantial improvement.

D-SCRIPT uses the model developed by Bepler & Berger [54], which is essentially a Bi-LSTM (bidirectional long short-term memory) neural network trained on protein information. However, Bi-LSTM-based models might fail to capture direct relationships between the feature vectors and the result label [56], causing a lower performance compared to the other models’ performance. Unlike D-SCRIPT, models like those proposed by Jha and co-authors [31] (GAT/GCN), SpatialPPIv2 [30], and PT-PPI use more complex transformer-based models (ProstT5 [52], SeqVec [57], ProtBert [58], and ESM-2 [59]) for embedding protein sequences. This allows them to process long sequences, avoiding the vanishing gradient problem common in LSTM architectures, make use of the self-attention mechanism [50] to integrate complex patterns across long protein dependencies, and generate context-aware embeddings at different scales. These implementations certainly show that transformer-based models pre-trained on large protein datasets significantly improve the prediction accuracy of DL models.

Among multimodal and graph-based approaches, SpatialPPIv2 [30] exhibited superior performance on the test set compared to the GAT and GCN models. This upgrade can be attributed to the manner in which SpatialPPIv2 constructs protein graphs and the enhanced implementation of GAT layers. Whereas Jha and co-authors [31] constructed graphs by connecting atoms based on their spatial proximity using atomic coordinates, SpatialPPIv2 constructs edges using a distance matrix within proteins. Furthermore, the authors incorporated a fictional element into the input proteins to enhance message passing among paired structures. This peculiarity allows the attention mechanism to calculate the weights dynamically and rely on features rather than on fixed graph structures like the ones we encountered with the models GCN and GAT.

Struct2Graph [32] showed the best performance among the graph-based models. Unlike GCN and GAT models, where the graph features generated by the GAT/GCN layers are pooled to obtain a fixed-size representation and passed to a fully connected layer to derive the final representation of the protein pairs, Struct2Graph implements a mutual attention network before concatenating the graph features. This module allows the model to extract relevant information about how the protein pairs mutually contribute to the interaction, and to generate a representative output vector used for calculating the predictions.

Despite the good performance of GSMFormer-PPI [33] trained on a subset of the PINDER dataset [35], we highlight that PT-PPI offers complementary methodological and biological strengths. From a methodological perspective, whereas GSMFormer-PPI integrates precomputed MaSIF surface descriptors with traditional graph-based representations of proteins, PT-PPI skips the pre-computation by adopting the fast-sampling algorithm of dMaSIF [22]. Moreover, it uses the SCHull [34] method for constructing sparse and connected graphs without dependence on arbitrary distance thresholds. Through this design, PT-PPI learns directly from raw point cloud representations of protein surfaces, utilizing the self-attention of Point Transformer layers.

In contrast, while GSMFormer-PPI was designed to focus on residue-level and a patched representation of the protein surfaces, PT-PPI points to the local geometry and the inherently unordered structural organization of the molecular surface. Remarkably, both models converge on the importance of protein surfaces as key regions that regulate physical contact and recognition between proteins and other molecules. Each model addresses the challenge from a distinct angle; however, both capture complementary levels of structural and physicochemical detail.

While adopting a simplified architecture and demanding a significantly lower preprocessing cost, PT-PPI demonstrated competitive predictive performance, underscoring the significance of geometric and surface-based representations in encoding the determinant features of protein interaction. Consequently, PT-PPI should be regarded not merely as a direct competitor of GSMFormer-PPI, but rather as a complementary approach that offers additional scalability for large-scale interactome analysis.

Independent of the good performance of graph- and multimodal-based models, PT-PPI consistently surpasses these architectures by a notable margin, achieving higher ROC AUC and PR AUC values. The model’s high PR AUC value reflects robustness in identifying true interactions under class imbalance, a common challenge in PPI datasets. These findings suggest that integrating surface information, sequential embeddings, and well-constructed sparse and connected graphs enables attention-based architectures, such as Point Transformers, to learn more discriminative features for PPI prediction. Collectively, it offers a promising direction for future developments in structure-informed PPI prediction, with potential applications in drug discovery and large-scale interactome mapping.

### 3.3 Assessing importance of SCHull method

We designed four baseline models employing conventional graph construction methodologies, including radial cutoff (r=3 Å and r=16 Å) and k-NN (k=8 and k=16), to evaluate the benefit of using SCHull as a method for generating geometric graphs in our framework. The generated graphs were used to train, validate, and evaluate the PT-PPI model under identical dataset size, hyperparameter, and training configurations to ensure a fair comparison. We additionally performed a 3-fold cross-validation on the training set using these designed baselines. Table 6 summarizes the results of the cross-validation. The performance of these baselines on the test set, relative to PT-PPI, is presented in the Table 7. A detailed description of the parameters used for creating the baseline graphs is available in the Supplementary Materials.

**Table 6:** Cross-validation results of PT-PPI using conventional graphs.

Model	Accuracy	Precision	Recall	F1 Score	AUROC	AUPRC
radius 3	0.963 ± 0.0009	0.954 ± 0.0016	0.972 ± 0.0010	0.963 ± 0.0010	*0.994*± 0.0001	0.993 ± 0.0003
radius 10	0.962 ± 0.0007	0.951 ± 0.0040	0.973 ± 0.0028	0.962 ± 0.0007	*0.994*± 0.0001	0.993 ± 0.0001
kNN 8	0.962 ± 0.0011	0.952 ± 0.0032	0.973 ± 0.0012	0.962 ± 0.0012	*0.994*± 0.0003	0.993 ± 0.0004
kNN 16	0.961 ± 0.0002	**0.956**± 0.0040	0.966 ± 0.0044	0.961 ± 0.0002	*0.994*± 0.0001	0.993 ± 0.0001
**PT-PPI with SCHull**	**0.966**± 0.0018	0.945 ± 0.0036	**0.988**± 0.0018	**0.966**± 0.0019	*0.994*± 0.0001	**0.994**± 0.0007

**Table 7:** Assessing the importance of the SCHull method via baseline models. Best results are in bold.

Model	Accuracy	Precision	Recall	F1 Score	AUROC	AUPRC
radius 3	0.864	0.852	0.881	0.866	0.934	0.921
radius 10	0.862	0.843	0.889	0.866	0.930	0.915
kNN 8	0.846	0.871	0.813	0.841	0.931	0.916
kNN 16	0.851	0.857	0.844	0.850	0.927	0.911
PT-PPI	**0.940**	**0.937**	**0.943**	**0.940**	**0.983**	**0.979**

Our findings demonstrate that implementing the SCHull method to construct geometric graphs from protein point cloud representations significantly enhances the performance of the point transformer-based layers in our proposed PT-PPI model. The cross-validation experiments indicate that all graph-construction methods yielded good performance and low standard deviation across folds, demonstrating that the results are highly stable and not sensitive to random variation. PT-PPI with SCHull graphs exhibits some fluctuation in variance compared to fixed-radius or k-NN graphs. Nevertheless, this is expected due to the adaptive and parameter-free nature of the method.

The evaluation metrics on the test set using graphs generated via radial cutoff or k-NN at two different scales were significantly comparable across the four experiments and validations, indicating that these strategies can benefit the representation of geometric objects such as molecules and proteins. However, since they rely on parameters like the number of neighbours or the threshold distance between points, it can lead to the problems we described before. To face these limitations, we emphasise the importance of methodologies that eliminate the possibility of isolated or disconnected nodes. For this purpose, the SCHull algorithm offers a robust alternative since it guarantees that the convex hull of a finite set of points $Z=z_{j}$ on a sphere is always connected, and that the number of edges is bounded above by three times the number of nodes, hence ensuring sparsity and connectivity.

### 3.4 Performance of PT-PPI on Baranwal’s dataset

To evaluate the performance of our model on data from a different distribution, and to determine whether it can be biased by the presence of samples derived only from the PINDER [35] dataset, we trained and tested PT-PPI using an independent dataset derived from the study of Baranwal and co-authors [32] in their model Struct2Graph. This dataset is characterised by the presence of direct/physical interactions of folded protein globules, excluding weakly interacting and loosely associated biomolecules. The samples were obtained by compiling information from curated databases such as IntAct and STRING, storing protein interactions of several model organisms, e.g, *Saccharomyces cerevisiae, Homo sapiens Escherichia coli, Caenorhabditis elegans and Staphylococcus aureus*. Furthermore, this dataset selected negative samples derived from the large-scale two-hybrid experiments of Trabuco and co-authors [60], with an extra cross-validation with the IntAct and STRING pairs to confirm that only the pairs that are not involved in any interaction at all were chosen. The final dataset comprises 4698 positive, 5036 negative samples (10.004 pairs). We ran a 3-fold cross-validation with random seeds on the train test, and tested the model on the held-out test set. Table 8 summarizes the performance of PT-PPI after 3-fold cross-validation. We report the average of each metric across all the runs and calculate its respective standard deviation. The performance of the held-out PINDER and Baranwal’s test sets is also reported.

**Table 8:** Performance of PT-PPI on Baranwal’s [32] and PINDER’s [35] test set.

Model	Accuracy	Precision	Recall	F1 Score	AUROC	AUPRC
Averaged CV	0.8407 ± 0.0155	0.8283 ± 0.0256	0.8672 ± 0.0313	0.8466 ± 0.0134	0.9178 ± 0.0108	0.9264 ± 0.0099
PT-PPI on Baranwal’s test	0.795	0.79	0.825	0.807	0.885	0.901
PT-PPI on PINDER’s test	**0.940**	**0.937**	**0.943**	**0.940**	**0.983**	**0.979**

The cross-validation results show that PT-PPI performs consistently across folds on the independent dataset, indicating that the model learns valuable information from protein surfaces and sequences. At the same time, it retains high discriminative ability even on small and heterogeneous datasets, as it is evidenced by AUROC and AUPRC scores over 90 %. On the other hand, the evaluation performed on the held-out test set demonstrates a PT-PPI model predicting new interactions with competitive accuracy and evaluation scores. There is no doubt that PINDER and Baranwal’s dataset differ in several aspects, such as, the size, diversity of protein families, and different strategies for sampling negatives which represents a challenge for DL models, and therefore leads to a reduction on the performance. Nevertheless, these scores also confirm that our model is not overfitting on PINDER, since metrics remain competitive, and can even be improved by increasing the dataset size.

Overall, this experiment reflects the potential of PINDER as a high-quality dataset for training DL models that rely on surface and structural representations. Specialized and smaller datasets derived from other curated databases can be used as an alternative for finetuning the trained models and further enhancing their robustness and generalization.

### 3.5 Ablation studies

To understand the contribution of individual components within the PT-PPI model, we conducted a series of ablation studies assessing performance following removal of specific features, including protein sequence embeddings, the global positional encoder, and the PT block. The positional encoding δ was removed to evaluate the effect on the performance of the model while ablating the global spatial information between neighbouring points. Similarly, the ablation of the PT block removes the relative positional vector, $\Delta_{ij}$, allowing us to clarify the contribution of the geometric self-attention. The PT module was replaced by an MLP for processing the geometric graphs with the protein surface features. All the parameters used before remained the same, as well as the dataset and splits.

Additionally, we evaluated PT-PPI under varying architectural depths, testing configurations with up to five point transformer layers, as the number of layers is a critical hyperparameter in deep learning models. Insufficient or excessive network depth can adversely affect accuracy, potentially resulting in under- or overfitting in complex architectures [61]. Table 9, Table 10 summarize the averaged metrics and their respective standard deviation after we ran a 3-fold cross-validation with independent seeds, and the performance metrics of PT-PPI on the test set under different experimental configurations, respectively.

**Table 9:** Cross-validation results of ablation experiments.

Model	Accuracy	Precision	Recall	F1 Score	AUROC	AUPRC
W/O sequence embeddings	0.914 ± 0.0065	0.887 ± 0.0102	0.923 ± 0.0048	0.914 ± 0.0055	0.962 ± 0.0025	0.950 ± 0.0029
W/O positional encoder	0.918 ± 0.0072	0.931 ± 0.0227	0.861 ± 0.0090	0.909 ± 0.0078	0.966 ± 0.0021	0.968 ± 0.0020
W/O PT block	0.902 ± 0.0058	0.938 ± 0.0125	0.855 ± 0.0108	0.894 ± 0.0059	0.958 ± 0.0018	0.961 ± 0.0020
PT-PPI 1 layer	0.895 ± 0.0080	0.880 ± 0.0095	0.892 ± 0.0083	0.886 ± 0.0082	0.950 ± 0.0030	0.941 ± 0.0035
**PT-PPI 2 layers**	**0.966**± 0.0018	**0.945**± 0.0036	**0.988**± 0.0018	**0.966**± 0.0019	**0.994**± 0.0005	**0.994**± 0.0007
PT-PPI 3 layers	0.959 ± 0.0021	0.938 ± 0.0041	0.981 ± 0.0022	0.958 ± 0.0020	0.992 ± 0.0007	0.991 ± 0.0010
PT-PPI 4 layers	0.955 ± 0.0025	0.934 ± 0.0038	0.978 ± 0.0026	0.955 ± 0.0023	0.991 ± 0.0009	0.990 ± 0.0011
PT-PPI 5 layers	0.932 ± 0.0045	0.914 ± 0.0068	0.958 ± 0.0049	0.935 ± 0.0042	0.984 ± 0.0017	0.978 ± 0.0022

**Table 10:** Performance of the PT-PPI model under different configurations. Best results are in bold.

Model	Accuracy	Precision	Recall	F1 score	AUROC	AUPRC
W/O sequence embeddings	0.908	0.881	**0.944**	0.911	0.959	0.948
W/O positional encoder	0.911	**0.966**	0.853	0.906	0.964	0.967
W/O PT block	0.899	0.941	0.851	0.894	0.957	0.959
PT-PPI 1 layer	0.854	0.856	0.851	0.853	0.935	0.919
**PT-PPI 2 layers**	**0.940**	0.937	0.943	**0.940**	**0.983**	**0.979**
PT-PPI 3 layers	0.908	0.884	0.938	0.910	0.961	0.950
PT-PPI 4 layers	0.913	0.912	0.914	0.913	0.966	0.955
PT-PPI 5 layers	0.780	0.745	0.854	0.795	0.836	0.816

The results reveal a slight reduction in performance metrics when sequence embeddings are excluded from the model input. However, the performance is relatively high, indicating that the model continues to perform well even in the absence of sequential information. This underscores the critical role of geometric and chemical surface features in the prediction of PPI using attention-based architectures such as point transformers. Similarly, the positional encoder was removed from the input of the point transformer architecture to assess whether the surface features alone are sufficient for the model to make accurate predictions. PT-PPI continued to perform well without explicit 3D positional information, suggesting that although spatial positions contribute to performance, the learned surface geometric features are more relevant for the model.

Ablation studies on the number of point transformer layers further clarified the optimal network depth for balancing accuracy and computational efficiency. Our findings indicate that a model with two layers is optimal for predicting accurate outcomes with the available dataset. Reducing the depth to a single layer caused a marked drop in accuracy, indicating that the model, while still able to capture basic information, fails to learn the hierarchical structure of protein surfaces. In contrast, increasing the number of layers does not result in improved performance. The predictive power of these models is significantly diminished beyond three layers, suggesting the necessity for more robust regularization strategies, data augmentation techniques, or pre-training to enhance their performance.

## 4 Conclusion

In this work, we propose PT-PPI as a new framework for the prediction of PPI. Our model integrates point cloud surface representations, sparse and connected geometric graphs derived from the hyper-parameter-free method SCHull, and the Point Transformer architecture. PT-PPI captures both local and global structural dependencies by modelling the protein surfaces as oriented point clouds enriched with geometric and chemical features. The performance of the model with SCHull graphs as input, in comparison to the performance with radial-cutoff and k-NN graphs at different scales, demonstrates that the SCHull method eliminates hyperparameter dependence in graph construction. This ensures both connectivity and sparsity, which are critical for efficient and robust deep learning on oriented point cloud representation of protein structures. The results of the ablation experiments further confirm the joint importance of sequence embeddings, positional encodings, and the depth of transformer layers for accurate predictions. It also highlights the need to optimize how we exploit the heterogeneity of protein representations, with specific emphasis on surface representation.

We acknowledge that several challenges remain to be addressed. Subsequent research should explore larger and more diverse protein complexes to validate the robustness of the methodology across species and environmental conditions.

Despite the high predictive accuracy of our model, enhancing its interpretability through the visualization of predicted interaction interfaces would offer deeper biological insights. The built-in attention and SCHull analysis tools in PT-PPI provide a direct means to visualize and interpret the key surface features driving its interaction predictions; however, a comprehensive interpretability study remains a task for future work. Further directions also include extending the framework beyond binary classification to different biological tasks such as binding affinity prediction, as well as integrating other state-of-the-art protein language models to further enrich the sequence representations. To summarize, the results presented here demonstrate that deep learning on protein surfaces, empowered by the Point Transformer architecture and SCHull graphs, is a powerful perspective to gain a deeper understanding of how proteins interact with other proteins and biomolecules.

## CRediT authorship contribution statement

**David Arteaga:** Writing – review & editing, Writing – original draft, Validation, Investigation, Formal analysis, Conceptualization. **Maria Poptsova:** Writing – review & editing, Supervision, Project administration, Methodology, Investigation, Funding acquisition, Conceptualization.

## Funding

The work was supported by the grant for research centers in the field of AI provided by the Ministry of Economic Development of the Russian Federation in accordance with the agreement 000000С313925P4E0002 and the agreement with HSE University no. 139-15-2025-009.

## Declaration of competing interest

The authors declare that they have no known competing financial interests or personal relationships that could have appeared to influence the work reported in this paper.

## Data Availability

The source code to train the model and replicate our results is available at https://github.com/bdabykov/PT_PPI
