# Supplementary material for: Prediction of protein-protein interactions using point transformer and spherical Convex Hull graphs
Source: Comput Struct Biotechnol J. 2025 Dec 16;31:82–93. doi: 10.1016/j.csbj.2025.12.008 (PMC12795690; doi:10.1016/j.csbj.2025.12.008)
Supplement: Multimedia Component 1 [file mmc1.pdf]

Supplementary Materials for Paper: Multimodal Graph, Surface, and Language-Based Model for Protein Protein Interaction Prediction

Arteaga B.D., Chervov, N.R, Poptsova M.S.

MaSIF's computational costs and parameters.

For the implementation of MaSIF (Gainza *et al*., 2020) we used the Docker version provided by the authors here, (See Docker container). This version immediately loads all the software prerequisites and dependencies required.

We ran the MaSIF pipelines on a HPC Cluster with OS CentOS 7.9, and GPU NVIDIA Tesla V100 32 GB. The tasks were parallelly computed using the job scheduling system SLURM, with batches of 1000 pairs and slots of 20-50 pairs in one run.

For a pair of proteins where the chain L has 105 residues, 1597 atoms, and the chain R contains 124 residues, 1946 atoms the precomputation steps will take 181,82 seconds. As was reported by the authors in the original paper (supplementary Fig. 12), in average, MaSIF takes about 2 minutes to pre-process one protein with length ranging between 110-200 amino acid residues. This computation time includes the following steps: computing surfaces, input features, and coordinates, decomposing into patches, and computing MaSIF-search descriptors.
